# Identification of avian wax synthases

**DOI:** 10.1186/1471-2091-13-4

**Published:** 2012-02-04

**Authors:** Eva-Maria Biester, Janine Hellenbrand, Jens Gruber, Mats Hamberg, Margrit Frentzen

**Affiliations:** 1 Institute of Biology I, RWTH Aachen University, (Worringer Weg 1), Aachen, (52074), Germany; 2 Department of Medical Biochemistry and Biophysics, Karolinska Institute, (Scheeles Vag 2), Stockholm, (17176), Sweden

## Abstract

**Background:**

Bird species show a high degree of variation in the composition of their preen gland waxes. For instance, *galliform *birds like chicken contain fatty acid esters of 2,3-alkanediols, while *Anseriformes *like goose or *Strigiformes *like barn owl contain wax monoesters in their preen gland secretions. The final biosynthetic step is catalyzed by wax synthases (WS) which have been identified in pro- and eukaryotic organisms.

**Results:**

Sequence similarities enabled us to identify six cDNAs encoding putative wax synthesizing proteins in chicken and two from barn owl and goose. Expression studies in yeast under *in vivo *and *in vitro *conditions showed that three proteins from chicken performed WS activity while a sequence from chicken, goose and barn owl encoded a bifunctional enzyme catalyzing both wax ester and triacylglycerol synthesis. Mono- and bifunctional WS were found to differ in their substrate specificities especially with regard to branched-chain alcohols and acyl-CoA thioesters. According to the expression patterns of their transcripts and the properties of the enzymes, avian WS proteins might not be confined to preen glands.

**Conclusions:**

We provide direct evidence that avian preen glands possess both monofunctional and bifunctional WS proteins which have different expression patterns and WS activities with different substrate specificities.

## Background

Birds preen their feathers with a secretion produced by the uropygial gland, a holocrine bilobular gland located above their tail. The secretion consists of lipids, proteins and salts [1] and varies, for example, among species, age, season and sex [2-7]. These secretions confer different functions regarding sexual attraction, lubrication, waterproofing, antipathogenic effects and plumage maintenance [8-11]. Preen gland waxes show a high diversity of components; some species contain monoacyl esters, others diacyl esters or triacylglycerols (TAG). The distribution of fatty acids and alcohol residues is often unique, especially branched-chain, extremely long-chain or substituted fatty acids can be found here [12]. For instance, the preen gland secretions from chicken (*Gallus gallus*) consist of 50% wax diesters and 30% TAG [13]. Wax diesters contain erythro- and threo-alkane-2,3-diols with chain-lengths of 21 to 23 carbon atoms and saturated fatty acids of 12 to 20 carbon atoms [13,14]. Diester waxes are detected in other *galliform *birds as quail or pheasant as well as perching birds, pigeons, cranes or woodpeckers [12,15-17], although wax monoesters are the most frequently found components of preen gland secretions. *Anseriformes *like geese (*Anser domesticus*) contain wax monoesters in their preen gland secretion, in which di-, tri- or tetramethylated acyl groups are esterified with saturated straight-chain monoalcohols [11]. In goose 96% of the alcohol component is due to octadecanol, while the fatty acid residues consist of 76% 2,4,6,8-tetramethyldecanoic acid and 11% 2,4,6,8-tetramethylundecanoic acid [18]. In contrast to the wax monoesters of geese, those found in barn owl (*Tyto alba*) are rich in methyl-branched fatty alcohol and fatty acyl residues. About 60% of the components are monomethyl-branched, mainly 3-methyl-branched C13 or C17 acids and 2-, 3- or 4-methyl-branched fatty alcohols with 12 to 18 carbon atoms [19].

Production of wax esters has already been observed in preen gland membranes of chicken [20] and goose [21]*in vitro*, so it could be assumed, that genes essential for wax ester biosynthesis are expressed in preen gland tissue. The respective genes have not been identified in birds yet, but wax ester synthase sequences (acyl-CoA:alcohol acyltransferases, AWAT, WS) have already been described in other organisms including mammals [22,23], plants [24-27], bacteria [28-30] and protozoa [31]. Mammalian enzymes with wax synthase activity have been found within members of both DGAT1 and DGAT2 type acyltransferase families [22,32]. DGAT (acyl-CoA:diacylglycerol acyltransferases) catalyze the final step in storage lipid biosynthesis of TAG, but the human DGAT1 is capable of synthesizing wax monoesters, diesters and retinylesters as well [32,33]. Human wax synthases AWAT1 and AWAT2 belong to the DGAT2 type family. Like DGAT1, AWAT2 is a multifunctional acyltransferase which shows *in vitro *acyl-CoA:monoacylglycerol acyltransferase (MOGAT), DGAT, WS and retinylester synthase activities [33]. Bacterial wax synthases are at least bifunctional enzymes conferring WS activity next to DGAT and low MOGAT activity [28,29,34].

Wax esters are excellent lubricants because of their high stability under high temperature and pressure and high resistance to hydrolysis [35]. Unlike saturated long-chain monoesters, mono-unsaturated monoester or diester waxes combine good lubricity with good thermal and oxidative stability, high viscosity indices [36] and stability against lipases [37]. To achieve the renewable production of wax esters, currently attempts are made to identify new enzymes catalyzing respective esterification reactions [22,25,31,38]. Production of wax esters in oil crops [35,39] or microorganisms [38,40] might in future be able to surrogate fossil materials in technical industry.

Our studies identified WS genes of chicken, goose and barn owl, members of different bird families with distinct preen wax compositions. As the chicken genome was fully sequenced and assembled in March 2004 by the National Human Genome Research Institute [41,42], it served as a starting point for the identification of avian WS genes. Several sequences were successfully cloned and functionally analyzed in yeast cells.

## Results

### Identification of putative wax synthases from avian organisms

Sequence similarity based searches conducted with human AWAT1, AWAT2 and DGAT1 sequences as queries against the annotated *Gallus gallus *proteome resulted in five full-length sequences. Using mRNA isolated from preen glands as starting material, we succeeded in cloning the respective cDNAs of GgWS2 [NCBI: JQ031644], GgWS4 [NCBI: XP_419207.1], GgWS5 [NCBI: NP_0010261921.1] and GgDGAT1 [NCBI: JQ031642], while cDNAs of GgWS1 were synthesized because we failed to amplify the respective full-length sequence. Based on the WS sequences from chicken, we were able to clone WS4 and WS5 from preen glands of barn owl and goose.

The cloned 1056 bp GgDGAT1 sequence showed almost 90% identity with the annotated mRNA in databases [NCBI: XM_422267.2, ENSEMBL: ENSGALT00000006691], its central region, however, differed from both annotations. On protein level, GgDGAT1 displayed 98% identity to a recently identified sequence from turkey [NCBI: XP_003208594] but differed from both annotations of chicken (Additional File 1).

The 1089 bp open reading frame (ORF) of GgWS2 differed from the annotations [NCBI: XM.426251.2, ENSEMBL: ENSGALT00000006967], especially in the 3'-terminal 60 nucleotides, while an alternation in the 5'-region causes a conservative substitution of V66I only (Additional File 2).

Unlike GgWS2, the GgWS4 ORF of 951 bp resembled that deposited at NCBI database [NCBI: XM_419207.2] except for two conservative nucleotide substitutions (T300C and T312C), which did not alter the amino acid sequence [NCBI: XP_419207.1]. Comparison with the respective sequences from goose [NCBI: JQ031643] and barn owl [NCBI: JQ031645] showed about 90% identity at the cDNA level and 94% sequence identity at the protein level (Additional File 3).

WS5 homologs from chicken [NCBI: NM_001031021], goose [NCBI: JQ031647] and barn owl [NCBI: JQ031646] were identical at the nucleotide level. Contrasting the NCBI sequence, we found two nucleotide substitutions, T299C and T317G, which led to the exchange of V100A and V124G on protein level [NCBI: NP_001026192.1].

### *In silico *analysis of putative avian wax synthases

**Figure 1:**
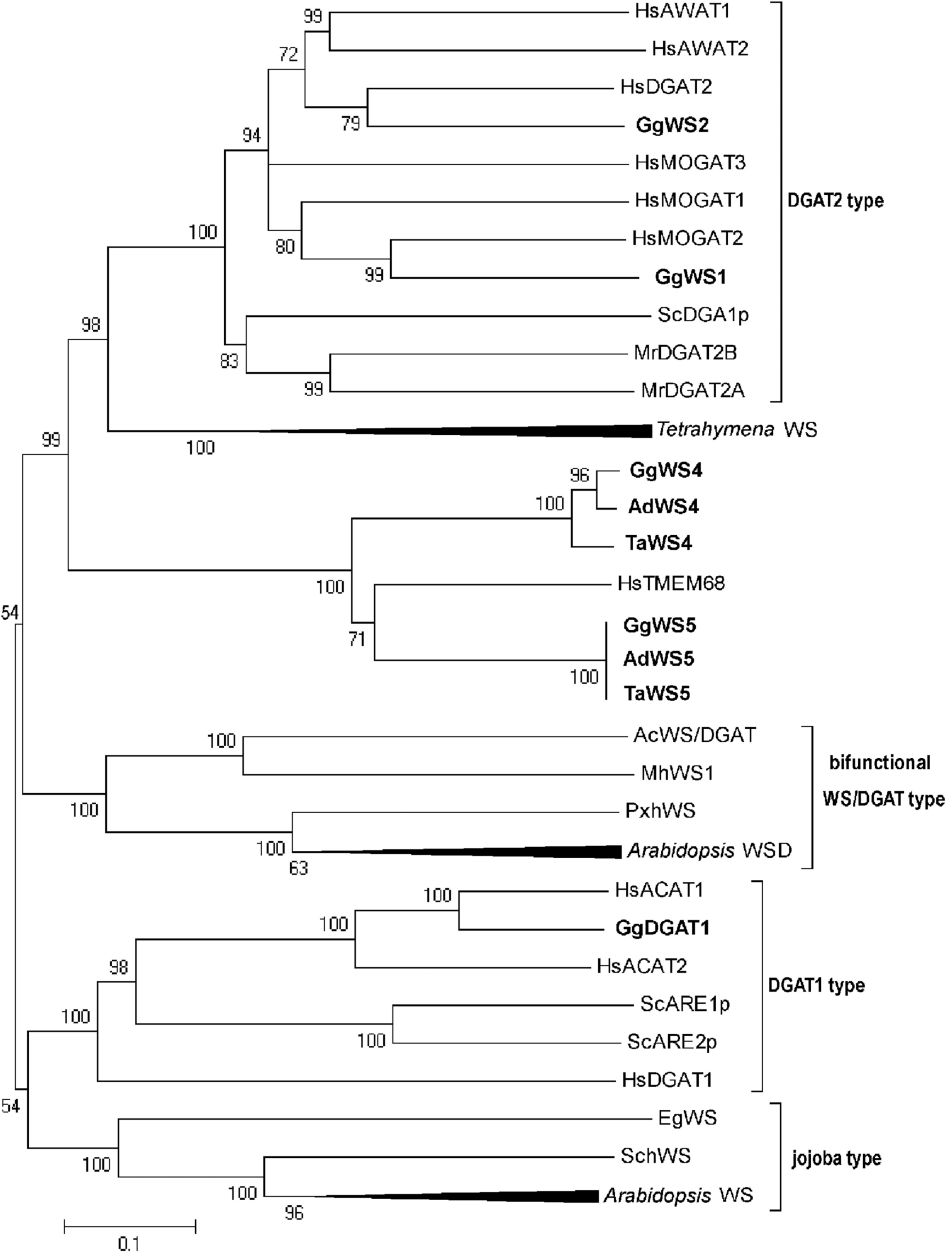
**Phylogenetic analysis of putative avian wax synthases in comparison to different acyltransferases from human, plants, yeast, *Tetrahymena *and bacteria**. The phylogram shows the relation of the analyzed avian proteins with acyltransferases from different organisms. The tree is based on the alignment of the following sequences (NCBI accession numbers are given): HsAWAT1 [*Homo sapiens*, NP_001013597.1], HsAWAT2 [*H. sapiens*, NP_001002254.1], HsMOGAT3 [*H. sapiens*, NP_835470], HsMOGAT2 [*H. sapiens*, NP_079374], HsMOGAT1 [*H. sapiens*, NP_477513.2], HsACAT1 [*H. sapiens*, NP_003092.4], HsACAT2 [*H. sapiens*, NP_003569.1], HsDGAT2 [*H. sapiens*, NP_115953.2], HsDGAT1 [*H. sapiens*, NP_036211.2], SchWS [*Simmondsia chinensis*, AF149919_1], EgWS [*Euglena gracilis*, ADI60058.1], PxhWS [*Petunia x hybrida*, AAZ08051.1], AcWS/DGAT [*Acinetobacter *sp. ADP1, YP_045555.1], GgWS1 [*Gallus gallus*, XP_424082.2], GgWS2 [*G. gallus*, JQ031643], GgWS4 [*G. gallus*, XP_419207.1], GgWS5 [*G. gallus*, NP_001026192.1], AdWS5 [*Anser domesticus*, JQ031647], TaWS5 [*Tyto alba*, JQ031646], AdWS4 [*A. domesticus*, JQ031643], TaWS4 [*T. alba*, JQ031645], HsTMEM68 [*H. sapiens*, Q96MH6.2], GgDGAT1 [*G. gallus*, JQ031642], *Tetrahymena *WS [*Tetrahymena thermophila*, XP_001027910, XP_001026090, XP_001008104, XP_001019739], MhWS1 [*Marinobacter hydrocarbonoclasticus*, ABO21021.1], *Arabidopsis *WSD [*Arabidopsis thaliana*, NP_568547.1, NP_177356.1, NP_850307.1, NP_200151.2] MrDGAT2B [*Umbelopsis ramanniana*, AAK84180.1], MrDGAT2A [*U. ramanniana*,AAK84179.1], ScDGA1p [*Saccharomyces cerevisiae*, NP_014888.1], ScARE1p [*S. cerevisiae*, CAA42296.1], ScARE2p [*S. cerevisiae*, CAA96298.1], *Arabidopsis *WS [*A. thaliana*, NP_200345.1, XP_002866091.1, NP_200349.1, NP_200346.1]. The scale corresponds to amino acid substitutions per site in the alignment of 41 sequences with a total of 188 positions. Numbers at the branches are bootstrap values indicating the probability of this relationship in %. Values above 95 can be regarded as correct. The dendrogram was created with ClustalX2 and MEGA5 software.

Comparison of the avian WS protein sequences with respective sequences from different organisms gave the results illustrated in Figure 1. It reveals that GgWS1 and GgWS2 possess the highest similarity to human acyltransferases of the DGAT2-type (up to 60%) while GgDGAT1 shows the highest identity to DGAT1 family members, namely 62% to human acyl-CoA:cholesterol acyltransferase (ACAT) HsACAT1, 43% to HsACAT2 and 16% to HsDGAT1. Avian WS4 and WS5 proteins share less than 15% sequence identity to both DGAT1 and DGAT2 family members and build an own branch of enzymes (Figure 1). WS4 and WS5 proteins comprise 55% identity to each other and WS5 proteins are most similar to transmembrane protein 68, a protein of unknown function found in various organisms (Figure 1).

**Figure 2:**
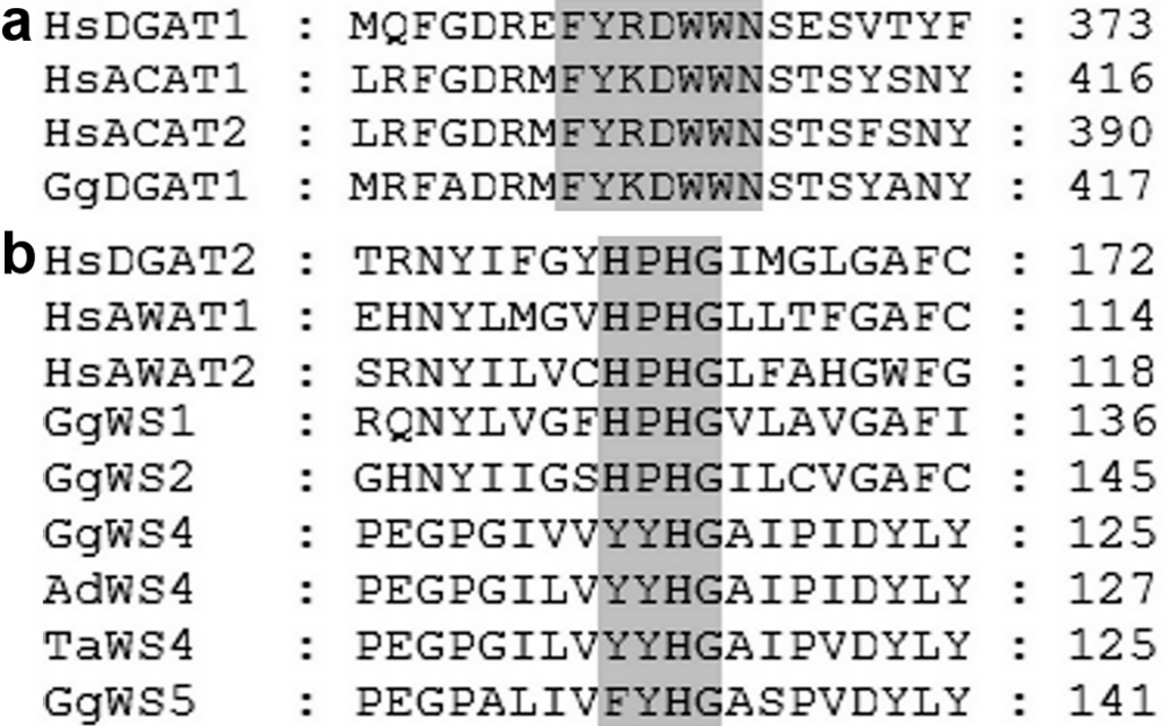
**Alignment of typical motifs of DGAT1 (a) and DGAT2 (b) families**. Figure 2a shows the alignment of the FYxDWWN motif which is a potential acyl-CoA binding motif in proteins of the DGAT1 family [44]. Figure 2b represents the partially modified HPHG motif representing a potential part of the active site in DGAT2 family proteins [45]. NCBI accession numbers of reference proteins: HsDGAT1 [*Homo sapiens*, NP_036211.2], HsACAT1 [*H. sapiens*, NP_003092.4], HsACAT2 [*H. sapiens*, NP_003569.1], HsDGAT2 [*H. sapiens*, NP_115953.2], HsAWAT1 [*H. sapiens*, NP_001013597.1], HsAWAT2 [*H. sapiens*, NP_001002254.1].

The relation to the different mammalian acyltransferase families was reflected in further characteristics of the avian proteins like the molecular mass, the transmembrane structure and acyltransferase motifs. Conserved domain search revealed that all DGAT1 family members possessed an MBOAT (membrane bound O-acyltransferase) superfamily motif (Pfam cl00738) and the FYxDWWN motif (Figure 2a), which is a predicted acyl-CoA binding site identified in mammalian DGAT1 family members [43,44]. Furthermore, GgDGAT1 and the human ACAT proteins contained the (H/Y)SF motif [43] which might play a role in sterol binding. Unlike DGAT1 family members, DGAT2 family members like GgWS1 and GgWS2 were found to contain an acyltransferase superfamily motif (Pfam cl00357), that was found in WS4 and WS5 as well. In addition, the HPHG motif which is typical for DGAT2 family members and likely comprises a part of the active site [45] was also found in GgWS1 and GgWS2, while in WS4 and WS5 homologs the motif was modified to YYHG and FYHG, respectively (Figure 2b). GgWS1 and GgWS2 have masses of about 40 kDa with one predicted N-terminal transmembrane domain (TMD) and their C-terminal parts stretched into the cytosol like other DGAT2 related proteins. On the other hand, GgDGAT1 is a 60 kDa protein with several predicted TMDs typical of DGAT1 related proteins. With regard to the transmembrane structure, the avian WS4 homologs resemble DGAT2 family members unlike WS5 homologs, which differed insofar as the TMD prediction indicated that the C-terminal part of the protein was not located in the cytosol.

### Expression profiles of wax synthase sequences in chicken

**Figure 3:**
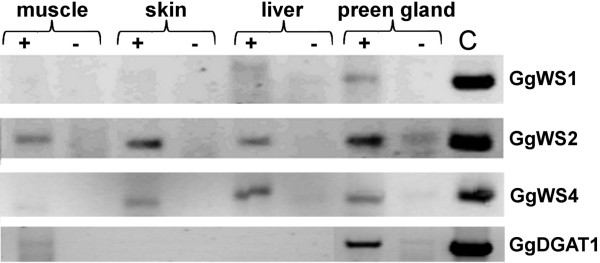
**Expression profiles of avian WS sequences in different tissues of chicken**. RNA was converted to cDNA with gene specific primers and used as template for partial transcript amplification. Reactions were performed with or without (+ and - marks) reverse transcriptase (C, positive control with plasmid DNA).

Expression profiles of four chicken sequences were analyzed with different chicken tissues to reveal whether the sequences were preferentially expressed in preen glands. As the use of oligo(dT) primers did not lead to the amplification of any wax synthase sequence, gene-specific primers were used to synthesize the respective cDNAs from 1 μg of RNA. The comparison of products from the different tissues indicated that GgWS1 and GgDGAT1 were almost exclusively expressed in the preen gland, while GgWS2 and GgWS4 were expressed in the four analyzed tissues (Figure 3).

### Functional expression in yeast

**Figure 4:**
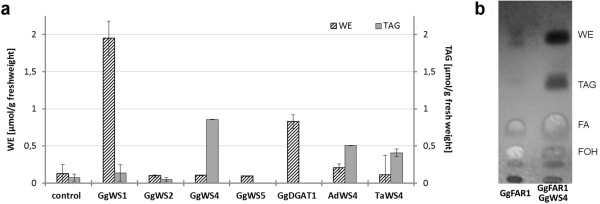
**Production of lipids in transgenic yeast cultures**. Cultures expressing one of the respective WS sequences were supplemented with a mixture of 125 μM 10:0-OH, 12:0-OH, 14:0-OH, 16:0-OH and 18:0-OH and induced for 48 hours. Lipids of the harvested cells were extracted and analyzed. Mean values and standard deviations of two independent experiments are given (a). TLC analysis of lipid extracts from yeast cells expressing GgFAR1 or co-expressing GgFAR1 and GgWS4 from cultures supplemented with 14:0-fatty acid (b). (FA: fatty acids, FOH: fatty alcohols, TAG: triacylglycerols, WE: wax esters)

To analyze the identities of the avian proteins, the respective cDNAs were expressed in a yeast mutant strain lacking TAG synthesis. Transgenic yeast cultures were supplemented with fatty alcohols from 10 to 18 carbon atoms to enable wax ester production. Lipid analyses of the transgenic yeast cells suggest that most of the avian proteins were functionally expressed and caused accumulation of storage lipids in significant but different levels and patterns (Figure 4a). Expression of GgWS1 resulted in the highest levels of wax esters which comprised 2 μmol wax ester/g fresh weight, while TAG was formed in very low levels only (Additional file 4). GgDGAT1 expressing yeast cells produced almost 1 μmol wax esters/g but no TAG (Additional file 5), whereas all WS4 homologs catalyzed the synthesis of higher levels of TAG (250 to 500 nmol/g) than of wax esters (100 to 250 nmol/g). The produced wax esters contained mainly dodecanol (12:0-OH) and tetradecanol (14:0-OH) esterified with palmitoleic (16:1) and oleic (18:1) acid irrespective of the expressed sequence. In contrast, TAG produced by WS4 homologs consisted of almost equal amounts of saturated and unsaturated fatty acyl residues. GgWS2 expressing cells contained storage lipids in very low levels similar to those of the control cells (Additional file 4). Additional feeding of GgWS2 expressing yeast cells with myristic acid (14:0) gave a 5-fold increase in wax production while the wax level of control strains was not affected. Such stimulation was also observed in GgWS4 expressing cells (Additional File 6).

To reconstitute the avian wax biosynthesis in yeast cells, WS were co-expressed with the recently identified fatty acyl-CoA reductase GgFAR1 [46]. This enzyme has a specificity for 16:0-acyl-chains, but is also able to produce 14:0-OH in yeast cultures supplemented with 14:0-FA [46]. GgWS4 was chosen for co-expression because it performed the highest WS activities of all analyzed WS enzymes with long chain acyl-acceptors. Supplementation with 14:0-FA of cultures expressing both GgWS4 and GgFAR1 resulted in the production of fatty alcohols, 350 nmol TAG/g and 550 nmol wax esters per g fresh weight, while expression of GgFAR1 alone led only to the production of fatty alcohols (Figure 4b).

### Properties of avian proteins

To analyze the substrate specificities of the different avian proteins, *in vitro *assays were performed with membranes of yeast cells expressing the respective sequences as enzyme source. WS activity was detectable in membranes, but not in soluble fractions, which is in line with the predicted transmembrane domains. Under standard assay conditions the WS activities were constant for at least 2 μg protein in an incubation time of 20 minutes at 35°C (data not shown). This holds true with regard to yeast membranes harboring GgWS1, GgWS2 and the WS4 proteins. Unlike GgWS5, which did not show acyltransferase activities under any conditions and with any substrates tested, GgDGAT1 was catalytically active in yeast cells, but not in isolated yeast membranes (Additional File 7). Addition of detergents, divalent cations or bovine serum albumin did not improve but partially inhibited incorporation rates of the labeled 16:0-acyl-groups into lipophilic reaction products (data not shown).

**Figure 5:**
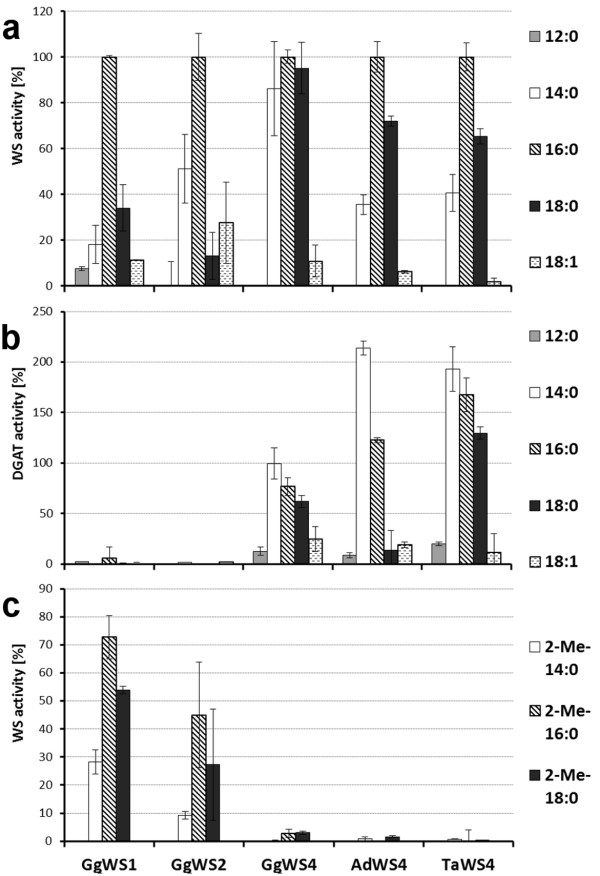
**Acyl-CoA specificities of avian enzymes**. Relative WS (a and c) and DGAT (b) activities of isolated yeast membranes from yeast cells expressing an avian WS sequence. Assays were conducted with 10:0-OH and the given straight-chain (a and b) or branched-chain (c) acyl-CoA thioesters under standard assay conditions (a and b) or increased protein amount (10 μg) and incubation time (2 h) (c). The relative activities of each protein are given, 100% corresponds to the activities with 10:0-OH and 16:0-CoA under identical conditions. Values are mean values from at least two independent assay series. (GgWS1: 116 pmol*min^-1^*mg^-1^; GgWS2: 105 pmol*min^-1^*mg^-1^; GgWS4: 149 pmol*min^-1^*mg^-1^; AdWS4: 126 pmol*min^-1^*mg^-1^; TaWS4: 149 pmol*min^-1^*mg^-1^).

GgWS1, GgWS2 and the avian WS4 proteins showing WS activity were further analyzed concerning their reaction products and their acyl-donor and -acceptor specificities. As given in Figure 5, GgWS1 and GgWS2 formed wax esters as main reaction products, while WS4 homologs possessed both WS and DGAT activities. These results which were in line with yeast expression experiments (Figure 4) clearly demonstrate the mono- or bifunctionality of the respective avian WS (Additional File 7).

With regard to the acyl-CoA specificities, avian enzymes were most active with saturated acyl-CoA thioesters of 14 to 18 carbon atoms, while the WS and DGAT activities were rather low with shorter or unsaturated thioesters. GgWS1 and GgWS2 were almost exclusively active with 16:0-CoA while the avian WS4 homologs produced the highest wax amounts with 16:0- or 18:0-CoA and the highest TAG levels with 14:0-CoA (Figure 5a and 5b). Hence bifunctional WS, especially those of goose and barn owl, displayed different acyl-CoA specificities regarding their WS and DGAT activities.

In addition to straight-chain acyl-donors, we assayed 2-methyl-branched acyl-CoA thioesters of 14 to 18 carbon atoms as especially barn owls contain many methyl-branched fatty acids in their preen gland secretion [19]. To detect WS activity with these substrates we had to increase the protein amount and incubation time. Under these conditions GgWS1 and GgWS2 showed appreciable activities with branched-chain acyl-CoA in comparison to 16:0-CoA, while activity of WS4 proteins were very low in comparison to 16:0-CoA (Figure 5c). GgWS1 displayed the highest activity with 2-methyl-branched 16:0-CoA that comprised 70% of the activity determined with 16:0-CoA under the same conditions. GgWS2 performed lower WS activities than GgWS1 but the substrate specificities of both enzymes were very similar to each other (Figure 5c).

**Figure 6:**
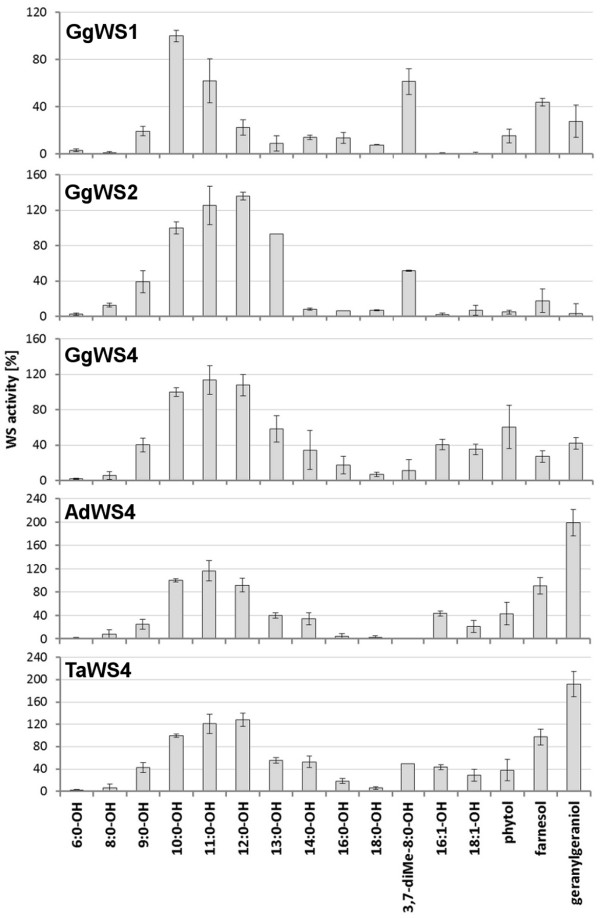
**Acyl-acceptor specificities of avian proteins**. Relative WS activities of membrane fractions of yeast cells harboring an avian enzyme with 16:0-CoA and the given acyl-acceptors under otherwise standard conditions. 100% corresponds to the activity with 16:0-CoA and 10:0-OH (GgWS1: 116 pmol*min^-1^*mg^-1^; GgWS2: 105 pmol*min^-1^*mg^-1^; GgWS4: 149 pmol*min^-1^*mg^-1^; AdWS4: 126 pmol*min^-1^*mg^-1^; TaWS4: 149 pmol*min^-1^*mg^-1^), values are mean values from at least two independent assay series.

**Figure 7:**
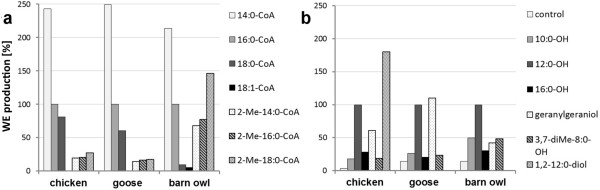
**Properties of WS activities of preen gland membranes of chicken, goose and barn owl**. Relative WS activities of preen gland membranes with 12:0-OH and different acyl-CoA thioesters (a) or 16:0-CoA and different alcohols (b). 100% corresponds to the turnover of 12:0-OH with 16:0-CoA. Standard assays were used apart of higher protein amounts (50 μg) and incubation times (2 h).

Analysis of acyl-acceptor specificities showed that all proteins performed the highest WS activities with decanol (10:0-OH) or undecanol (11:0-OH) in combination with 16:0-CoA, whereas no activities could be observed with alcohols shorter than 9 carbon atoms (Figure 6). While the enzymes showed very similar activity patterns with saturated alcohols of 9 to 16 carbon atoms, assays with unsaturated or branched-chain alcohols revealed differences in the substrate specificities of the enzymes. Unsaturated alcohols were no suitable substrates for GgWS1 or GgWS2 while the WS4 proteins used 16:1-OH and 18:1-OH with higher activities than saturated fatty alcohols of the same chain length. With regard to branched-chain substrates, we analyzed 3,7-dimethyl-octanol (3,7-diMe-8:0-OH) and isoprenols as substrates. Although octanol (8:0-OH) was not esterified by any enzyme, the 3,7-diMe-8:0-OH was esterified with high activities by all enzymes except GgWS4 and AdWS4. Isoprenols were converted to prenyl esters by AdWS4 and TaWS4 proteins, while chicken proteins showed low prenyl ester synthase activities only. Among the WS4 enzymes, geranylgeraniol was the preferred alcohol of TaWS4 and AdWS4 in contrast to the respective chicken protein (Figure 6). As diesters are the naturally occurring waxes in chicken preen glands [13,14], we analyzed 1,2- and 1,12-isomers of dodecanediol as substrates. None of the avian enzymes was capable of esterifying these diols, although activity could be detected in assays with membranes isolated from chicken preen glands (Figure 7).

In summary, we have identified both monofunctional and bifunctional WS enzymes from birds, which differ in their substrate specificities, especially with regard to branched-chain substrates.

### *In vitro *analysis of WS activities in preen gland membranes

To analyze the *in vitro *WS activities of preen glands, assays were performed with isolated membrane preparations. Strong WS activity could be detected in membranes of chicken preen glands, but comparably low activities were obtained in skin or liver preparations, suggesting that WS activity is restricted to preen gland tissue.

Figure 7a displays the acyl-donor specificities of avian WS using preen gland membranes of chicken, goose and barn owl as enzyme source. The highest WS activities were obtained with 14:0-CoA and almost no activity with 18:1-CoA. In contrast to chicken and goose, barn owl performed high activities with 2-methyl-branched acyl-CoA thioesters as well (Figure 7a).

The comparison of different acyl-acceptors proved that membranes of preen glands of all tested species were able to catalyze the esterification of straight-chain alcohols, especially 12:0-OH, or branched-chain alcohols like geranylgeraniol or 3,7-diMe-8:0-OH (Figure 7b). In addition to WS, membranes of chicken preen glands exhibited strong DGAT activity and also high diester synthase activity with 1,2-dodecanediol.

## Discussion and Conclusion

In this study we present the identification and characterization of the first avian wax synthase sequences. Although WS activity has already been demonstrated *in vitro *for cell free preparations from preen glands of chicken and turkey [47], neither a specific enzyme catalyzing this reaction nor the respective gene has been identified yet. Six of nine proteins identified by sequence homologies catalyzed wax ester syntheses *in vivo *or *in vitro *(GgDGAT1, GgWS1, GgWS2, GgWS4, AdWS4 and TaWS4) and WS4 homologs additionally showed DGAT activity.

With most avian proteins, *in vivo *and *in vitro *experiments gave consistent results. WS activity of GgDGAT1, however, was clearly detectable in yeast cells (Figure 4), but not in enzymatic assays with yeast membranes, in spite of enzymatic assays being more sensitive than feeding experiments. Human ACAT1, to which GgDGAT1 displays the highest sequence identity, was functionally expressed in yeast cells and showed *in vitro *ACAT activity with cholesterol and oleate [43], but GgDGAT1 did not show ACAT activity. Perhaps we have not tested GgDGAT1 under conditions suitable for this enzyme so far, or the chicken enzyme has a very low protein stability under *in vitro *conditions and is rapidly degraded by yeast proteases. This still remains to be determined.

The protein termed WS5 is highly conserved among vertebrates and identical in different bird species. It contains a lysophospholipid acyltransferase motif (cl00357 and cd07987), but the enzymatic activities have not been studied in any organism to date. Our experiments could not confirm any acyltransferase activity *in vivo *or *in vitro*.

All avian WS are found to be most active with saturated medium-chain alcohols (10:0-OH to 12:0-OH) and saturated long-chain acyl-CoA thioesters (14:0 to 18:0), but they differ in their reaction products and their activities with certain substrates. GgWS4, like the respective homologs from goose and barn owl, are bifunctional enzymes which catalyze both WE and TAG biosynthesis. They are more active with unsaturated than with saturated long-chain alcohols and effectively utilize branched-chain alcohols like isoprenols as acyl-acceptors, especially AdWS4 and TaWS4, but they show hardly any activities with branched-chain acyl-CoA. In contrast to the WS4 homologs, GgWS1 and GgWS2 are monofunctional enzymes which are inactive with saturated and unsaturated alcohols of more than 14 carbon atoms (Figure 6), but are active with methyl-branched alcohols and branched-chain acyl-CoA thioesters (Figure 5c). The ability of the chicken enzyme to utilize branched-chain substrates was surprising, as these components are typical for wax esters of barn owl and goose preen glands, but not for those of chicken. Perhaps WS1 and WS2 homologs from barn owl and goose might be even more active with branched-chain substrates than the chicken enzymes. These homologs likely caused the relatively high WE formation rates determined in assays with preen gland membranes of barn owl (Figure 7), while the relatively low activities of preen gland membranes of goose might be due to the wax esters containing tetramethylated but not monomethylated acyl-groups like barn owl glands [18,19].

The secretion of chicken preen glands is rich in 2,3-diester waxes [14,20] and preen gland membranes of chicken, unlike those of goose and barn owl, effectively catalyze the esterification of 1,2-dodecanediol (Figure 7). On the other hand, diester synthase activities with 1,2-dodecanediol were not detected with transgenic yeast membranes harboring one of the various avian enzymes even when WS assays with yeast membranes were run under conditions identical to those with preen gland membranes. These data suggest that diester waxes might be formed by a WS in chicken which is unrelated to known WS classes or by a related protein, which could not be detected in yeast membranes so far, like GgDGAT1. Anyhow, chicken preen gland membranes displayed high monoester synthase activities (Figure 7), which is in line with previous findings [47] and supports our results of the WS enzymes expressed in yeast (Figures 5 and 6).

## Methods

### Identification of putative wax synthases

BLASTp (protein-to-protein Basic Local Alignment Search Tool) [48] studies were undertaken to investigate the predicted *Gallus gallus *proteome for putative wax synthases using the NCBI server [49]. Human wax synthase sequences HsAWAT1 [NCBI: NP_001013597] and HsAWAT2 [NCBI: NP_001002254] were used as query sequences for DGAT2 family members and human HsDGAT1 [NCBI: NP_036211] was used as query for DGAT1 like proteins. The obtained results were drawn back on to the originating mRNA sequences, which were used for generating cloning PCR primers.

### Vector construction

Preparation of mRNA, cDNA synthesis and cloning was performed as published previously [46]. Specific primer sequences were deduced from NCBI sequences (DGAT1-For, 5'-ATGGCAGGTGAAGACTGTGTAAG-3', DGAT1-Rev, 5'-TTACATCTGCACGTGACATGACCAC-3', WS2-For, 5'-CACCATGAAAACAATCATTGCAGCCTG-3', WS2-Rev, 5'-TCAACTCCTGGGCCATGTGG-3', WS4-For, 5'-CACCATGACCTACCTAAGCTACTTTGC-3', WS4-Rev, 5'-CTAATCACTTTTACAATGTTTATC-3', WS5-For, 5'-CACCATGATAGGTAGCAATGAATCC-3', WS5-Rev, 5'-TTAGTCTTCTTTCTGTCGTGTTTG-3'). GgWS1 was synthesized (GeneArt) and derived in Gateway^® ^compatible entry vectors. All sequences were cloned into yeast expression vector pYES-DEST52 (Invitrogen) and for co-expression of wax synthases with the chicken fatty acyl-CoA reductase GgFAR1 [46] the respective sequences were cloned into pAG425GAL vectors (Addgene plasmid 14203, Susan Lindquist). Cotransformation of pYES-DEST52 and pAG425GAL enabled the selection on medium lacking uracil and leucine. The respective destination vectors were transformed into *Saccharomyces cerevisiae *BY4741 Δlro1 Δdga1 (MATa, his3Δ1, leu2Δ0, met15Δ0, ura3Δ0, lro1-Δ::kanMX4, dga1-Δ::natMX4) [50].

### Analysis of WS expression profiles

Expression profiles were analyzed in tissues of chicken pectoral muscle, skin, liver and preen gland. 1 μg of total RNA was digested with DNase (Fermentas) to remove genomic DNA contamination, cDNA synthesis was conducted as described previously [46]. The following reverse primers were used for first strand synthesis: DGAT1-Rev, 5'-TTACATCTGCACGTGACATGACCAC-3', WS1-Rev, 5'-CTATATAAATTCGAGGTGACTGTCTTCTG -3', WS2-Rev, 5'-TCAACTCCTGGGCCATGTGG-3', WS4-Rev, 5'-CTAATCACTTTTACAATGTTTATC-3'. Negative controls without reverse transcriptase were performed and treated likewise. The following PCR was conducted using SupraTherm Taq polymerase (Genecraft). The amplified internal fragments were chosen to span introns, in case of residual chromosomal DNA. (GgDGAT1-exon6 for, 5'-TGCATGTTCTGTGCCACGGTT-3', GgDGAT1-exon12 rev, 5'-AAGTGAACCAAGCACCTGTGCA-3', 435 bp fragment length, GgWS1-exon4 for, 5'-TCTTCCACGCAAGAGGTATC-3', GgWS1-exon5 rev 5'-GTTGGCTTGTGCTTCTTCAG-3', GgWS2-exon4 for, 5'-TTGCCGTGCCTGAGGAGATG-3' 112 bp fragment length, GgWS2-exon5 rev, 5'-CTGGGAACAGCTCGGAGAAG-3', 190 bp fragment length, GgWS4-exon3 for, 5'-GCTCTAGATGCGAAAGTGCCCA-3', GgWS4-exon5 rev, 5'-GGAGGAGTGAATGGCAATCG-3'; 129 bp fragment length). Plasmid DNA was used as positive control. The PCR products were analyzed on 2% agarose gels in TAE buffer.

### Phylogenetic analysis and structure prediction

Sequence analyses were carried out using ClustalX2 [51] and GeneDoc [52] software. Phylograms were computed with MEGA5 [53] and neighbor-joining method [54] with 1000 bootstrap replicates using *p*-distance method. All gaps were deleted for computation of evolutionary distances.

Molecular mass and isoelectric points were calculated using ProtParam [55] on the ExPASy Server [56]. Transmembrane helices of avian proteins and mammalian wax synthases were predicted using TMHMM software [57-60]. Predictions were compared to Kyte Doolittle plots [61] with window parameters of 19 which revealed similar results. Acyltransferase superfamily motifs and putative acyl-acceptor binding pockets were discovered by NCBI conserved domain search [62,63].

### Functional analysis in yeast

The *S. cerevisiae *BY4741 Δlro1 Δdga1 strain deficient in TAG synthesis [50] was used for expression experiments. Transgenic yeast cells expressing an avian sequence in pYES-DEST52 vectors (Invitrogen) were cultivated in SD minimal medium containing 0.17% (w/v) yeast nitrogen base (MP Biomedicals), 0.068% (w/v) complete supplement medium without uracil and leucine, 0.5% NH_4_SO_4 _(w/v), 0.01% (w/v) leucine and 2% glucose for 24 h at 28°C. The cells of 50 ml cultures were induced with 2% galactose for 48 h and were supplemented with 125 μM decanol, dodecanol, tetradecanol, hexadecanol and octadecanol (Sigma Aldrich). Transgenic yeast cells harboring both pYES-DEST52-GgFAR1 and pAG425GAL-GgWS4 were cultivated in SD medium without uracil and leucine and supplemented with 250 μM myristic acid. Cells were harvested, washed and stored at -20°C.

Extraction of yeast cells was performed according to Bligh and Dyer [64]. The lipid extracts were separated by TLC on preparative TLC plates (Silica Gel 60 plates 0.5 mm thickness, Merck) in heptane/diethyl ether/acetic acid (90/30/1, v/v/v) and visualized under UV light after spraying with dichlorofluorescein (0.3% (w/v) dissolved in isopropanol) [65]. Myristoyl-dodecanoate (Sigma Aldrich) and TAG isolated from sunflower oil were used as standards.

### GC analysis of WE and TAG

Bands co-chromatographing with the WE and TAG standards were transmethylated in 0.5 M sulfuric acid and 3% dimethoxypropane in methanol for 1 hour at 80°C together with 250 nmol docosanoic acid as internal standard. Fatty acid methyl esters (FAMEs) and fatty alcohols were extracted with heptane, concentrated and analyzed via gas chromatography (GC) with flame ionization detection (FID). For quantification of WE the total amount of fatty alcohols in the fractions was summarized, for quantification of TAG the sum of FAMEs was divided by 3.

GC-FID analysis was carried out using the HP6890 gas chromatograph equipped with an OPTIMA225 column (Macherey & Nagel) (25 m length, 0.25 mm diameter, 0.25 μm film thickness). 1 μl of the extract was analyzed in splitless injection with N_2 _as carrier gas (constant flow, 0.9 bar pressure, total column flow 1 ml/min) and inlet and detector temperatures of 260°C. A temperature program was carried out starting at 120°C, 8°C/min to 144°C, 4°C/min to 240°C. Peaks were identified by comparison of the respective retention times with those of standard substances of different fatty alcohols and FAMEs (Sigma Aldrich).

### Synthesis of [1-^14^C]-labeled branched-chain fatty acids

[1-^14^C]2-Methyltetradecanoic, 2-methylhexadecanoic and 2-methyloctadecanoic acids were prepared by α-methylation of the corresponding [1-^14^C]-labeled fatty acids via the sequence carboxylic acid → acyl chloride → diazoketone → chloroketone → 2-methylcarboxylic acid essentially as described [66]. Purification by reversed-phase HPLC (solvent system, acetonitrile/water/acetic acid 85:15:0.01, v/v/v) afforded > 98% pure materials having a specific radioactivity of 0.622 GBq/mmol.

### Preparation of yeast membranes and *in vitro *wax synthase assay

Membrane preparation and WS assays were performed as described previously [67]. Briefly, transgenic yeast cells were harvested, washed in Tris-H_2_SO_4 _(50 mM, pH 7.6), frozen and disrupted. Cell supernatants were combined and sonicated, cell debris was sedimented (2,500 × g, 15 min and 4°C) and the membranes were isolated from the supernatant by high speed centrifugation (1 h, 140,000 × g, 4°C). The sedimented membranes were resuspended in Tris-H_2_SO_4 _buffer and stored in aliquots at -80°C. The protein concentration was determined [68].

WS activity was measured with unlabeled acyl-acceptors and labeled acyl-CoA-thioesters as outlined before [63]. The following acyl-CoA thioesters were used: [1-^14^C]-myristoyl-CoA and [1-C^14^]-stearoyl-CoA (Biotrend), specific activity 2.03 Bq/pmol, [1-^14^C]-palmitoyl-CoA and [1-^14^C]-oleoyl-CoA (Perkin Elmer), specific activity 2.22 Bq/pmol and 2.03 Bq/pmol, [1-^14^C]-decanoyl-CoA (0.09 Bq/pmol) and [1-^14^C]-dodecanoyl-CoA (0.7 Bq/pmol) and 2-methyl-acyl-CoA (14:0, 16:0 and 18:0) (0.62 Bq/pmol) kindly provided by Prof. Sten Stymne and members of his laboratory, SLU Alnarp, Sweden. The reaction mixture of standard assays consisted of 10 mM BIS-Tris-propane buffer (pH 9), 13 μM [1-^14^C]-labeled acyl-CoA and 300 μM acyl-acceptor (Sigma Aldrich) using 2 to 4 μg protein of total yeast membrane fractions as enzyme source. Acyl-acceptors were dissolved in heptane and evaporated to dryness in the reaction tubes before addition of further assay components. Incubation was carried out at 35°C for 20 minutes. Lipids were extracted, applied to TLC silica gel plates (Merck) and chromatographed in heptane/diethyl ether/acetic acid (90/20/1 v/v/v). The bands were visualized with the FLA-3000 bioimager system (Fujifilm) and quantified in a liquid scintillation counter LS 6500 (Beckman Coulter). DGAT activity was measured as by-product of WS activity without addition of DAG, endogenous substrates were used for TAG synthesis.

Before calculation of relative activities, background activities of membranes from yeast control strains were subtracted.

### WS assay with isolated preen gland membranes

Membranes from dissected preen glands were prepared as described before [21]. Briefly, the secretions were removed, the tissues were cut into small pieces, homogenized in NaK-buffer (0.1 M Na-K-phosphate buffer, pH 7.6, 0.5 mM DTT, 1 mM MgCl_2_) using an Ultra-Turrax three times for 15 seconds. Residual fragments were removed by centrifugation at 2,500 × g for 15 min and membranes were sedimented by ultracentrifugation at 140,000 × g for one hour. The membranes were resuspended in an appropriate volume of NaK-buffer. The protein content of the prepared membrane fractions was determined [68] and about 50 μg protein was used in the WS assay as outlined above, but incubation time was extended to 2 hours.

## Abbreviations

ACAT: acyl-CoA:cholesterol acyltransferase, retinyl ester synthase; CoA: Coenzyme A; DGAT: acyl-CoA:diacylglycerol acyltransferase; MOGAT: acyl-CoA:monoacylglycerol acyltransferase; ORF: open reading frame; TAG: triacylglycerols; TMD: transmembrane domain; WE: wax esters; WS: wax synthase

## Authors' contributions

EMB designed and carried out experiments, analyzed all data and drafted the journal. JH identified and cloned GgFAR1, participated in the design of experiments and data analyses and revised the manuscript. JG cloned GgDGAT1 and participated in lipid analyses. MH synthesized methyl-branched fatty acids and wrote the corresponding method section. MF coordinated the study, was involved in drafting the manuscript and revised the manuscript. All authors read and approved the manuscript.

## Supplementary Material

Additional file 1 **Amino acid alignment of GgDGAT with database sequences**. Amino acid alignment of GgDGAT1 [NCBI: JQ031642] and respective sequences from NCBI [XP_422267.2] and ENSEMBL [ENSGALT00000006691] database. The grey background highlights the different amino acids of the cloned protein and the predicted database sequences in the N-terminal region and the central part. Click here for file

Additional file 2 **Protein alignment of GgWS2 with database sequences**. Protein alignments of GgWS2 [NCBI: JQ031644] and respective protein sequences on NCBI [XP_426251.2] and ENSEMBL [ENSGALT00000006967] database. The grey background highlights the different amino acids between the cloned sequence and the predicted database sequences at the C-terminus and the I66V substitution. Click here for file

Additional file 3 **Protein alignment of WS4 homologs**. Protein sequence alignment of WS4 homologs from chicken [Gg, NCBI: XP_419207], goose [Ad, NCBI: JQ031643] and barn owl [Ta, NCBI: JQ031645] WS4. The grey background highlights the differences in the amino acid sequences of different avian WS4 proteins. Click here for file

Additional file 4 **GC analyses of transmethylated wax esters and triacylglycerols**. Lipids were extracted from transgenic yeast cells expressing the empty vector (control) or one of the avian proteins under standard conditions. WE and TAG were reextracted from TLC plates, transmethylated and analyzed by GC. (1) 10:0-OH, (2) 12:0-OH, (3) 14:0-OH, (4) 16:1-FAME, (5)16:0-FAME, (6) 16:0-OH, (7) 18:1-FAME, (8) 18:1-FAME, (9) 22:1-ME (internal standard) Click here for file

Additional file 5 **GC analyses of intact wax esters and transmethylated triacylglycerols from control yeast strains and yeast cultures expressing GgDGAT1**. The yeast cultures expressing the empty vector (control) or GgDGAT1 were cultivated under standard conditions, lipids were extracted and separated by TLC. WE were extracted from TLC and analyzed as intact WE. TAG were extracted, transmethylated and analyzed as methylester-derivatives. (1) 22:1-ME (internal standard in WE analysis, 30 nmol), (2) 26:1-WE, (3) 26:0-WE, (4) 28:1-WE, (5) 28:0-WE, (6) 30:1-WE, (7) 30:0-WE, (8) 32:1-WE, (9) 32:0-WE, (10) 16:1-ME, (11) 16:0-ME, (12) 18:1-ME, (13) 18:0-ME, (14) 22:0-ME (internal standard in TAG analysis, 30 nmol) Click here for file

Additional file 6 **Wax ester production of transgenic yeast cells expressing GgWS2 or GgWS4 under different conditions**. Yeast cultures expressing the empty vector (control), GgWS2 or GgWS4 were induced for 48 hours in SD-medium containing 125 μM 10:0-, 12:0-, 14:0-, 16:0- and 18:0-alcohol (A), 500 μM 14:0 alcohol and fatty acid (B) or 500 μM 14:0 and 16:0 alcohol and fatty acid (C). The lipids were extracted and analyzed by GC, the total WE-amounts per gram fresh weight are given. Click here for file

Additional file 7 **TLC analysis of lipophilic reaction products from WS assays with yeast membranes**. Assays were performed with 16:0-CoA and 10:0-OH under standard conditions using membranes of yeast cells expressing one of the respective sequences. Reaction products were extracted from the assays, separated by TLC and visualized using the FLA-3000 imaging system. The analysis is representative of several repetitions. Click here for file
