# Supplementary material for: Identification of avian wax synthases
Source: BMC Biochem. 2012 Feb 4;13:4. doi: 10.1186/1471-2091-13-4 (PMC3316144; doi:10.1186/1471-2091-13-4)
Supplement: Additional file 1 — Amino acid alignment of GgDGAT with database sequences. Amino acid alignment of GgDGAT1 [NCBI: JQ031642] and respective sequences from NCBI [XP_422267.2] and ENSEMBL [ENSGALT00000006691] database. The grey background highlights the different amino acids of the cloned protein and the predicted database sequences in the N-terminal region and the central part. [file 1471-2091-13-4-S1.PDF]

```
                     *        20         *        40         *        60         *        80         *       100
GgDGAT1 : MAGEDCVRKRPSGSTTTYKSPENEEMQRRPDGDRSFQNSSNGRVDVDHVITRKMQLIAEAEQLKPVFMKEVDSHFTEFVNSLVAKSALLDSSSSASLFPA : 100
NCBI    : MAGEDCVRKRPSGSTTTYKSPENEEMQRRPDGDRSFQNSSNGRVDVDHVITRKMQLIAEAEQLKPVFMKEVDSHFTEFVNSLVAKSALLDSSSSASLFPA : 100
ENSEMBL : -------------------------MQRRPDGDRSFQNSSNGRVDVDHVITRKMQLIAEAEQLKPVFMKEVDSHFTEFVNSLVAKSALLDSSSSASLFPA :  75

                     *       120         *       140         *       160         *       180         *       200
GgDGAT1 : SCSEKELHKAKALRAPPEHGKIFTARRSLLDELFEVSHIRTIYHMFIALLIVFILSTLLVDFIDEGRLVLGFDLLVYVFGKFPVVFCTWLCMFCATVIIP : 200
NCBI    : SCSEKELHKAKALRAPPEHGKIFTARRSLLDELFEVSHIRTIYHMFIALLIVFILSTLLVDFIDEGRLVLGFDLLVYVFGKFPVVFCTWLCMFCATVIIP : 200
ENSEMBL : SCSEKELHKAKALRAPPEHGKIFTARRSLLDELFEVSHIRTIYHMFIALLIVFILSTLLVDFIDEGRLVLGFDLLVYVFGKFPVVFCTWLCMFCATVIIP : 175

                     *       220         *       240         *       260         *       280         *       300
GgDGAT1 : YSLFSQWAQGYCSSSHRVIYSLFYGTLFTLFQTVGLGIGPTYVAISYALPPASRFIVILEQVRLVMKAHSFVRENVPRVLSSVKEKSSSVPIPRISQYLY : 300
NCBI    : YSLF-----------------------------------------------------LISQVRLVMKAHSFVRENVPRVLSSVKEKS--IPIPRISQYLY : 245
ENSEMBL : YSLFSLWMESVYDLKKQLFGCFFFFTLIFYCIN---------FSECVSRLKLHLLLVVLWMVRLVMKAHSFVRENVPRVLSSVKEKSSSVPIPRISQYLY : 266

                     *       320         *       340         *       360         *       380         *       400
GgDGAT1 : FLFAPTLIYRDNYPRNPMVRWGYVATKFAQVLGSLFYAYYIFVRLCIPQFRNSSQETFNLRGLVLCIFNSILPGVLILFLVFFAFLHCWLNAFAEMMRFA : 400
NCBI    : FLFAPTLIYRDNYPRNPMVRWGYVATKFAQVLGSLFYAYYIFVRLCIPQFRNSSQETFNLRGLVLCIFNSILPGVLILFLVFFAFLHCWLNAFAEMMRFA : 345
ENSEMBL : FLFAPTLIYRDNYPRNPMVRWGYVATKFAQVLGSLFYAYYIFVRLCIPQFRNSSQETFNLRGLVLCIFNSILPGVLILFLVFFAFLHCWLNAFAEMMRFA : 366

                     *       420         *       440         *       460         *       480         *       500
GgDGAT1 : DRMFYKDWWNSTSYANYYRTWNVVVHDWLYYYAYRDFLWFFGKKFKAAAMLSVFTVSAAVHEYVLSICFGFFYPVLFCLFMCFGMLFNFILNDRRKGPIW : 500
NCBI    : DRMFYKDWWNSTSYANYYRTWNVVVHDWLYYYAYRDFLWFFGKKFKAAAMLSVFTVSAAVHEYVLSICFGFFYPVLFCLFMCFGMLFNFILNDRRKGPIW : 445
ENSEMBL : DRMFYKDWWNSTSYANYYRTWNVVVHDWLYYYAYRDFLWFFGKKFKAAAMLSVFTVSAAVHEYVLSICFGFFYPVLFCLFMCFGMLFNFILNDRRKGPIW : 466

                     *       520         *       540         *
GgDGAT1 : NVIMWTSLFLGQGVIICLYSQEWYARQYCPAENPAFLDYLKPRSWSCHVQM : 551
NCBI    : NVIMWTSLFLGQGVIICLYSQEWYARQYCPAENPAFLDYLKPRSWSCHVQM : 496
ENSEMBL : NVIMWTSLFLGQGVIICLYSQEWYARQYCPAENPAFLDYLKPRSWSCHVQM : 517
```
