# Supplementary material for: Identification of avian wax synthases
Source: BMC Biochem. 2012 Feb 4;13:4. doi: 10.1186/1471-2091-13-4 (PMC3316144; doi:10.1186/1471-2091-13-4)
Supplement: Additional file 2 — Protein alignment of GgWS2 with database sequences. Protein alignments of GgWS2 [NCBI: JQ031644] and respective protein sequences on NCBI [XP_426251.2] and ENSEMBL [ENSGALT00000006967] database. The grey background highlights the different amino acids between the cloned sequence and the predicted database sequences at the C-terminus and the I66V substitution. [file 1471-2091-13-4-S2.PDF]

```
                   *        20         *        40         *        60         *        80         *       100
GgWS2    : MKTIIAACSQNLSGSRASLHAALRTLLAVPWPSQRDVRAWLQLLAVLQWVLSFLLLGPVTLVLLIYLVFTRFWPISALYLAWVIFDWDTPEKGGRRLPCL : 100
NCBI     : MKTIIAACSQNLSGSRASLHAALRTLLAVPWPSQRDVRAWLQLLAVLQWVLSFLLLGPVTLVLLVYLVFTRFWPISALYLAWVIFDWDTPEKGGRRLPCL : 100
ENSEMBL  : MKTIIAACSQNLSGSRASLHAALRTLLAVPWPSQRDVRAWLQLLAVLQWVLSFLLLGPVTLVLLVYLVFTRFWPISALYLAWVIFDWDTPEKGGRRLPCL : 100

                   *       120         *       140         *       160         *       180         *       200
GgWS2    : RRWSVWRHFRDYFPVKLVKTHDLSPGHNYIIGSHPHGILCVGAFCNFITGSTGFSELFPGIRPFLTTLAGNFRLPLFREYLMSGGLCPVTRRAIGHLLSK : 200
NCBI     : RRWSVWRHFRDYFPVKLVKTHDLSPGHNYIIGSHPHGILCVGAFCNFITGSTGFSELFPGIRPFLTTLAGNFRLPLFREYLMSGGLCPVTRRAIGHLLSK : 200
ENSEMBL  : RRWSVWRHFRDYFPVKLVKTHDLSPGHNYIIGSHPHGILCVGAFCNFITGSTGFSELFPGIRPFLTTLAGNFRLPLFREYLMSGGLCPVTRRAIGHLLSK : 200

                   *       220         *       240         *       260         *       280         *       300
GgWS2    : NGTGNAVAIVIGGAAESLSCSPGVTTLILKNRKGFVRMALQHGAFLVPSFSFGENELFRQVVFEEGSWMRAVQQRFQKMMGFAPCVFYGRGLTSVRSRGF : 300
NCBI     : NGTGNAVAIVIGGAAESLSCSPGVTTLILKNRKGFVRMALQHGAFLVPSFSFGENELFRQVVFEEGSWMRAVQQRFQKMMGFAPCVFYGRGLTSVRSRGF : 300
ENSEMBL  : NGTGNAVAIVIGGAAESLSCSPGVTTLILKNRKGFVRMALQHGAFLVPSFSFGENELFRQVVFEEGSWMRAVQQRFQKMMGFAPCVFYGRGLTSVRSRGF : 300

                   *       320         *       340         *       360         *
GgWS2    : LPYARPITTVVGEPVTVPKIEEPSSEVVDLYHGMYVRSLLKLFNDNKTKASSWRQKSTWPRS------------ : 362
NCBI     : LPYARPITTVVGEPVTVPKIEEPSSEVVDLYHGMYVRSLLKLFNDNKTKYGLSEGDELRIVASSWRQKSTWPRS : 374
ENSEMBL  : LPYARPITTVVGEPVTVPKIEEPSSEVVDLYHGMYVRSLLKLFNDNKTKYGLSEGDELRIV------------- : 361
```
