# Supplementary material for: Identification of avian wax synthases
Source: BMC Biochem. 2012 Feb 4;13:4. doi: 10.1186/1471-2091-13-4 (PMC3316144; doi:10.1186/1471-2091-13-4)
Supplement: Additional file 3 — Protein alignment of WS4 homologs. Protein sequence alignment of WS4 homologs from chicken [Gg, NCBI: XP_419207], goose [Ad, NCBI: JQ031643] and barn owl [Ta, NCBI: JQ031645] WS4. The grey background highlights the differences in the amino acid sequences of different avian WS4 proteins. [file 1471-2091-13-4-S3.PDF]

```
                 *        20         *        40         *        60         *        80         *       100
GgWS4 : MTYLSYFACMLEEWTAVEYLKKYFFHMVIISVTISAILVFFIVPLTILFFIYLTNVLLIIYQRNGEVKA--DPLSDVWDSARKTVASFWDIYARIWHGYE :  98
AdWS4 : MTYLSYFAYILEEWTAVEYLKKYFFHMVIISLTISAMLIFFIVPLTILFFIYLTNVLLLIYQRNDEVKAAKDPLSNVWDSARKTVASFWDIYARIWHGYE : 100
TaWS4 : MTYLSYFAYVLEEWIVVEYLKEYLFHVVIVSLTISAILVFFIVPLTILFFIYLTNILLLIYQRNSEVKA--DPLSDVWDSARKTIASFWDIYARIWHGYE :  98

                 *       120         *       140         *       160         *       180         *       200
GgWS4 : LHGVENLPEGPGIVVYYHGAIPIDYLYFLSRLFLWKKRLCLSVADHFVFRLPGLKLLLEVTGVMPGTREECLSALKNGHLVSISPGGVREALFSDESYQL : 198
AdWS4 : LHGVENLPEGPGILVYYHGAIPIDYLYFLSRLFLWKKRLCLSVADHFVFRLPGLKLLLEVTGVMPGTREECLSALKNGHLVSISPGGVREALFSDESYQL : 200
TaWS4 : LHGVENLPEGPGILVYYHGAIPVDYLYFLSRLFLWKKRLCLSVADHFVFRLPGLKLLLEVTGVMPGTREECLIALKNGHLVSISPGGVREALFSDESYQL : 198

                 *       220         *       240         *       260         *       280         *       300
GgWS4 : MWGNRKGFAQVALDAKVPIIPMYTQNVREGYRMFKERRFFRQLYETTRLPFTPPYGGLPVKFRTYIGEPIPYDPNITTDELVEKTKTAVQALIKKHQTIP : 298
AdWS4 : MWGNRKGFAQVALDAKVPIIPMYTQNVREGYRMFKERRFFRKLYESTRLPFTPPYGGLPVKFRTYIGEPIPYDPNITTDELVEKTKTAVQALIKKHQTIP : 300
TaWS4 : MWGNRKGFAQVALDAKVPIIPMYTQNVREGYRMFKERRFFRQLYESTRLPFTPPYGGLPVKFRTYIGEPIPYDPNITTEELVEKTKTAVQALISKHQTIP : 298

                 *
GgWS4 : GSIWKALLDRFDKHCKSD : 316
AdWS4 : GSIWKALMDRFDKHCKSD : 318
TaWS4 : GSIWKALLDRFDKHCKSD : 316
```
