# Supplementary figures and images for: Identification of avian wax synthases
Source: BMC Biochem. 2012 Feb 4;13:4. doi: 10.1186/1471-2091-13-4 (PMC3316144; doi:10.1186/1471-2091-13-4)

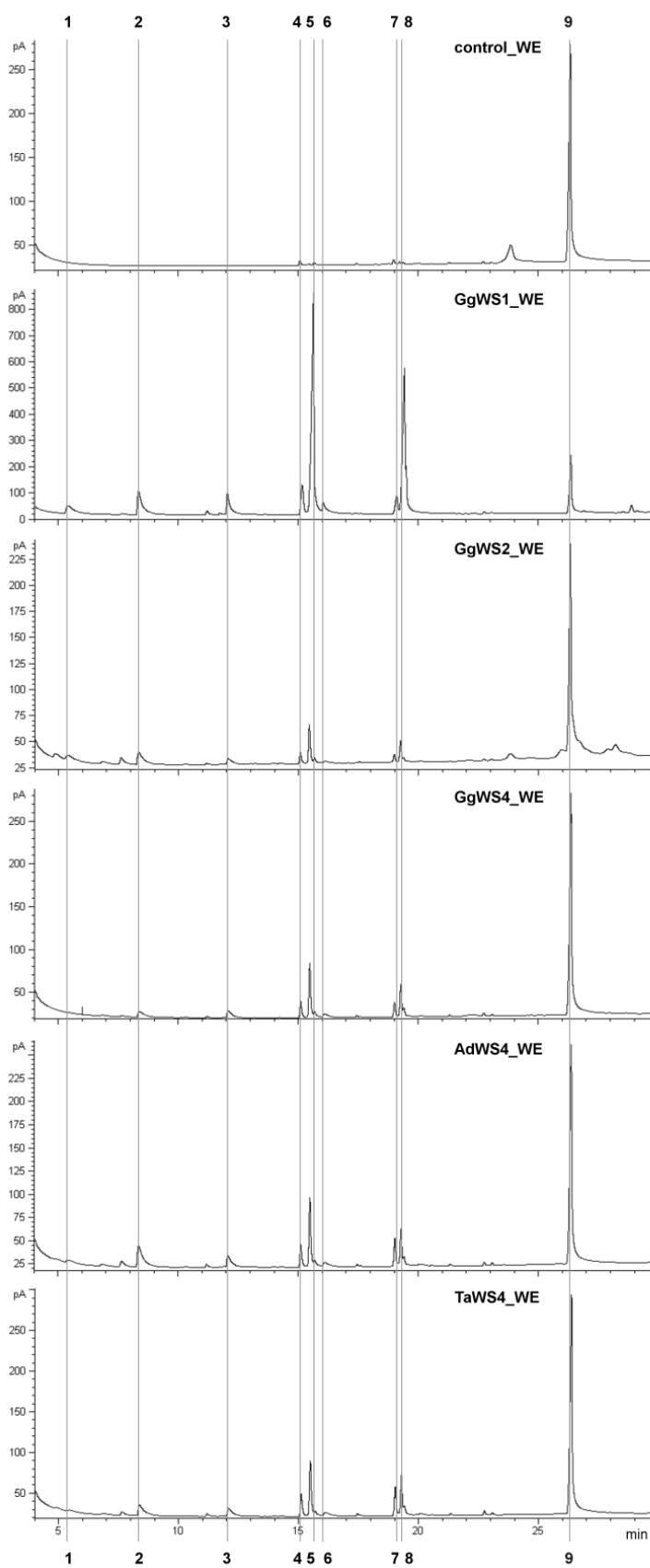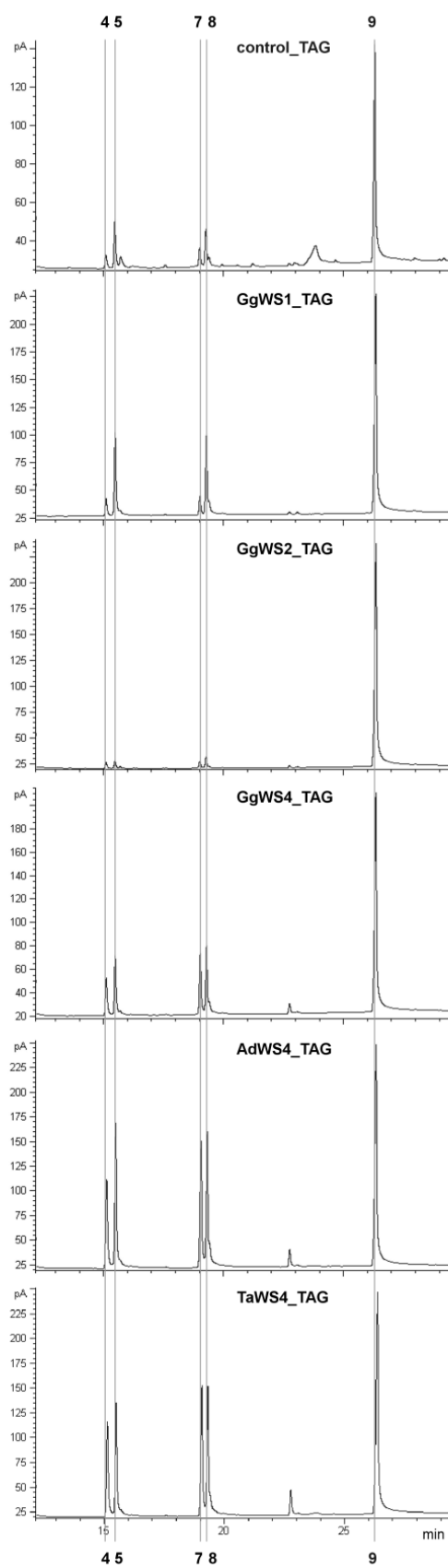

Supplement: Additional file 4 — GC analyses of transmethylated wax esters and triacylglycerols. Lipids were extracted from transgenic yeast cells expressing the empty vector (control) or one of the avian proteins under standard conditions. WE and TAG were reextracted from TLC plates, transmethylated and analyzed by GC. (1) 10:0-OH, (2) 12:0-OH, (3) 14:0-OH, (4) 16:1-FAME, (5)16:0-FAME, (6) 16:0-OH, (7) 18:1-FAME, (8) 18:1-FAME, (9) 22:1-ME (internal standard) [file 1471-2091-13-4-S4.PDF]

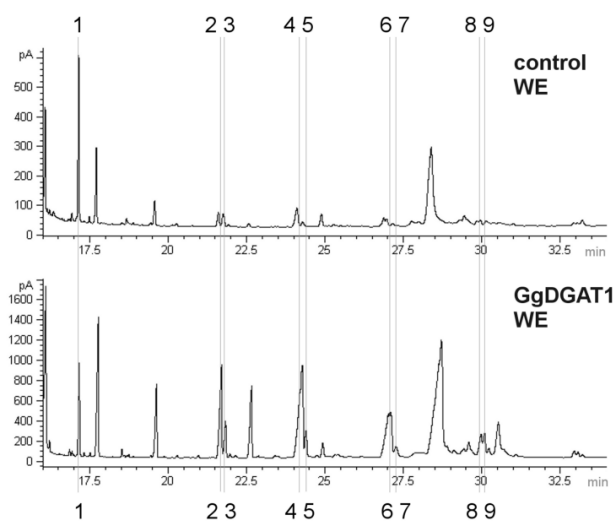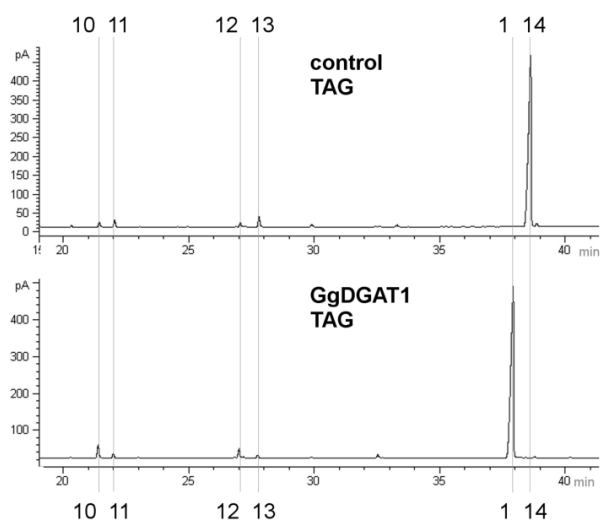

Supplement: Additional file 5 — GC analyses of intact wax esters and transmethylated triacylglycerols from control yeast strains and yeast cultures expressing GgDGAT1. The yeast cultures expressing the empty vector (control) or GgDGAT1 were cultivated under standard conditions, lipids were extracted and separated by TLC. WE were extracted from TLC and analyzed as intact WE. TAG were extracted, transmethylated and analyzed as methylester-derivatives. (1) 22:1-ME (internal standard in WE analysis, 30 nmol), (2) 26:1-WE, (3) 26:0-WE, (4) 28:1-WE, (5) 28:0-WE, (6) 30:1-WE, (7) 30:0-WE, (8) 32:1-WE, (9) 32:0-WE, (10) 16:1-ME, (11) 16:0-ME, (12) 18:1-ME, (13) 18:0-ME, (14) 22:0-ME (internal standard in TAG analysis, 30 nmol) [file 1471-2091-13-4-S5.PDF]

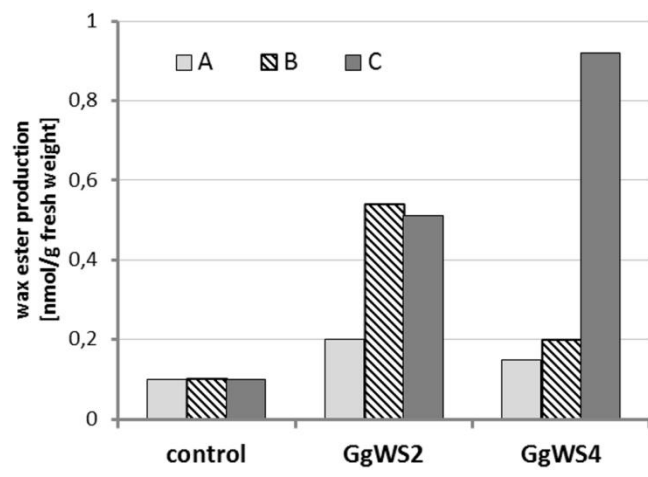

Supplement: Additional file 6 — Wax ester production of transgenic yeast cells expressing GgWS2 or GgWS4 under different conditions. Yeast cultures expressing the empty vector (control), GgWS2 or GgWS4 were induced for 48 hours in SD-medium containing 125 μM 10:0-, 12:0-, 14:0-, 16:0- and 18:0-alcohol (A), 500 μM 14:0 alcohol and fatty acid (B) or 500 μM 14:0 and 16:0 alcohol and fatty acid (C). The lipids were extracted and analyzed by GC, the total WE-amounts per gram fresh weight are given. [file 1471-2091-13-4-S6.PDF]

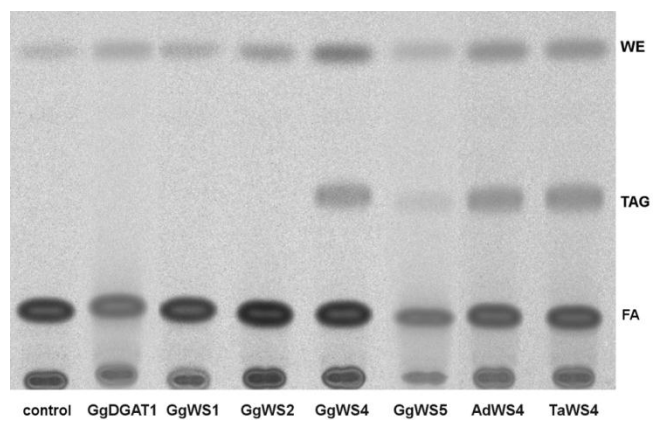

Supplement: Additional file 7 — TLC analysis of lipophilic reaction products from WS assays with yeast membranes. Assays were performed with 16:0-CoA and 10:0-OH under standard conditions using membranes of yeast cells expressing one of the respective sequences. Reaction products were extracted from the assays, separated by TLC and visualized using the FLA-3000 imaging system. The analysis is representative of several repetitions. [file 1471-2091-13-4-S7.PDF]
